# Cigarette smoking is associated with reduced neuroinflammation and better cognitive control in people living with HIV

**DOI:** 10.1038/s41386-024-02035-6

**Published:** 2025-01-01

**Authors:** Arthur L. Brody, Anna K. Mischel, Andre Y. Sanavi, Alvin Wong, Ji Hye Bahn, Arpi Minassian, Erin E. Morgan, Brinda Rana, Carl K. Hoh, David R. Vera, Kishore K. Kotta, Alannah H. Miranda, Nina Pocuca, Thomas J. Walter, Natalie Guggino, Renee Beverly-Aylwin, Jeffrey H. Meyer, Neil Vasdev, Jared W. Young

**Affiliations:** 1 Department of Psychiatry, University of California San Diego, San Diego, CA USA; 2 Department of Research, VA San Diego Healthcare System, San Diego, CA USA; 3 Department of Psychiatry, VA San Diego Healthcare System, San Diego, CA USA; 4 Department of Radiology, University of California San Diego, San Diego, CA USA; 5 Brain Health Imaging Centre, Azrieli Centre for Neuro-Radiochemistry, and Campbell Family Mental Health Research Institute, CAMH, Toronto, ON Canada; 6 Department of Psychiatry, University of Toronto, Toronto, ON Canada; 7 Present Address: Department of Research, VA San Diego Healthcare System, San Diego, CA USA

**Keywords:** Addiction, Human behaviour

## Abstract

People living with HIV (HIV+) are roughly twice as likely to smoke cigarettes (Smok+) as the general population. With the advent of effective antiretroviral therapies, it is increasingly important to understand the effects of chronic HIV infection and cigarette smoking on brain function and cognition since HIV+ individuals have heightened neuroinflammation and cognitive deficits even with such therapies. Based on prior studies demonstrating that smoking reduces a marker for neuroinflammation in HIV- individuals, we hypothesized that HIV+/Smok+ individuals would have less neuroinflammation and better cognitive control than HIV+/Smok- individuals. Fifty-nine participants (HIV-/Smok- [*n* = 16], HIV-/Smok+ [*n*=14], HIV+/Smok- [*n* = 18], and HIV+/Smok+ [*n* = 11]) underwent baseline eligibility tests, positron emission tomography (PET) scanning to determine levels of a marker for neuroinflammation, and assessment of cognitive control with the reverse-translated 5-choice continuous performance test (5C-CPT), with smokers having smoked to satiety prior to testing. For the PET data, a significant effect of smoking status on whole brain (WB) standardized uptake value (SUV) was found between HIV+/Smok+ and HIV+/Smok- participants (due to 18.8% lower WB SUV in the HIV+/Smok+ group). HIV+/Smok- participants exhibited a mean 13.5% higher WB SUV than HIV-/Smok- participants. For the 5C-CPT, HIV+/Smok+ participants performed significantly better than HIV+/Smok- participants (d prime), and HIV+/Smok- participants performed worse than HIV-/Smok- participants. Thus, HIV+/Smok+ individuals demonstrated lower levels of the neuroinflammation marker and better cognitive control than HIV+/Smok- individuals. Given that HIV+ individuals whose HIV is well-controlled can still have chronic neurocognitive complications, study results suggest possible paths for future research into nicotine-related treatments to prevent such complications.

## Introduction

People living with HIV (PLWH; HIV+) are living longer thanks to combination antiretroviral therapy (ART), moving HIV from a ‘fatal’ to ‘chronic’ disease [1]. Even with effective ART, complications still arise in PLWH, including impacts on health and cognitive functioning (e.g., attentional deficits), with up to half suffering from HIV-associated neurological disorders. Such deficits are driven in-part by poor cognitive control, where PLWH have difficulty maintaining goals while inhibiting distractions from such goals. Chronic inflammation, including neuroinflammation, occurs in PLWH [2] and may be a source of these detrimental health effects. Given that PLWH are roughly twice as likely as the general population to smoke cigarettes [3–5] and have more trouble quitting [3, 4], and that nicotine (a primary constituent of tobacco smoke) is anti-inflammatory [6] and pro-cognitive [7], understanding the effects of being HIV+ and cigarette smoking on neuroinflammation and cognitive control in PLWH is crucial. To our knowledge, there have been no published studies examining the effects of cigarette smoking on neuroinflammation and cognitive control in PLWH.

To examine neuroinflammation in vivo, radioligands such as ^18^F-labeled fluoroethoxybenzyl-*N*-(4-phenoxypyridin-3-yl) acetamide ([^18^F]FEPPA) have been developed that reliably label the translocator protein 18 kDa (TSPO) [8, 9] for positron emission tomography (PET) scanning. TSPO is a mitochondrial membrane protein that becomes more highly expressed when microglia become activated [10]. Microglia participate in functions central to neuroinflammation, (e.g., clearance of apoptotic cells and extracellular pathogens, removal of degenerating neurons and extracellular proteins, and cytokine/chemokine production [11]). TSPO radioligand binding correlates with the presence of activated microglia identified by immunohistochemistry in situ [12] and immunohistochemistry combined with autoradiography in brain tissue [9, 13], and is elevated in illnesses known to be associated with gliosis [14], though TSPO signal also arises from astroglia and endothelial cells [15]. Taken together, TSPO measurement levels with PET scanning can be used as a marker for neuroinflammation levels. [^18^F]FEPPA and other second generation radioligands have relatively high affinity for TSPO, enabling high-sensitivity for detecting TSPO availability and thus small group differences [8, 13, 16–18].

Multiple lines of evidence indicate that chronic HIV infection leads to neuroinflammation [2], including PET studies utilizing TSPO-labelling radioligands. Cognitively-healthy PLWH (stable on ART) and those with cognitive impairment consistently have elevated levels of TSPO compared to HIV- controls both globally and regionally [19–21]. Furthermore, increased TSPO binding in multiple brain regions (e.g., hippocampus, amygdala, and thalamus) has been associated with poorer cognitive performance [19, 21]. While these studies using newer radioligands with increased affinity for TSPO report differences in binding between PLWH and controls, earlier studies of this type [22, 23] using an older, first-generation TSPO radioligand with less specific binding did not. In addition to brain imaging studies, CSF studies revealed increased biomarkers for neuroinflammation in treated PLWH [24, 25]. Altogether, PLWH have chronically increased neuroinflammation, seen in both asymptomatic and cognitively-symptomatic people. To date, no effective treatment exists for cognitive deficits in PLWH; however, a potential inference can be drawn from the elevated prevalence of cigarette smoking in PLWH, possibly as self-medication.

A recent preclinical and clinical research review reported that nicotine has anti-inflammatory and neuroprotective effects, leading to mild cognitive enhancement [26]. Chronic nicotine administration reliably improves cognitive control in mice [27] and humans [28] as measured by continuous performance tests (CPTs), driven by improving target detection. Given that tobacco use has not been found to be a risk factor for neurocognitive impairment in PLWH [29], it is important to determine the impact of smoking on cognitive control in PLWH, particularly on target detection. While impaired decision-making (Iowa Gambling Task), impulsivity (Barratt Impulsiveness Scale) [30], working memory (N-back) [31], and learning and memory [32] have been observed in PLWH smokers relative to PLWH non-smokers, studies that take into account nicotine withdrawal (cognitive deficits arise within a few hours of stopping smoking [33, 34]) tend to report performance benefits [7]. Because impulsivity, indecision, and other symptoms tested in the preceding studies may occur during withdrawal [27, 35, 36], the potential confound of nicotine withdrawal requires consideration. The present study examined PLWH smokers who smoked to satiety immediately before testing to minimize withdrawal as a confound.

Prior indirect evidence supports the hypotheses that PLWH smoke to reduce neuroinflammation and improve cognitive control. For neuroinflammation, others have demonstrated elevated levels in PLWH compared to HIV- controls [19–21], while we demonstrated that HIV- cigarette smokers who smoked to satiety [37] or underwent overnight abstinence [38] had lower whole brain (WB) standardized uptake values (SUVs) on PET scanning than HIV- non-smokers (15.5–17.0% across studies), indicating reduced neuroinflammatory function from smoking. For cognitive control, we demonstrated that nicotine administration (primary active compound in smoking) enhanced this function in rodents, as measured by the 5-choice continuous performance test (5C-CPT) [27], which was also seen in humans [39].

For the present study, we had single a priori hypotheses for each of the two parts of the study, namely that PLWH who smoke would have: (1) lower WB SUV on PET scanning and (2) better cognitive control (as measured by the 5C-CPT) than PLWH who do not smoke. We also evaluated the specificity of this effect within PLWH by determining the impact of smoking in people without HIV, as controls. We hypothesized that effects of smoking on the marker for neuroinflammation would occur throughout the brain, since prior research by our group [37, 38, 40–43] and others [44, 45] demonstrates widespread effects of smoking when studying systems that are widely distributed. Thus, our a priori hypothesis for the neuroinflammation marker was the following rank order (from lowest to highest): [1] HIV-/Smok+, [2] HIV-/Smok- and HIV+/Smok+ (no statistical difference), [3] HIV+/Smok-. We further hypothesized that cognitive control (5C-CPT performance) would have the above rank order from best to worst.

## Methods

### Study overview

Fifty-nine otherwise healthy adults, who were in one of four categories, completed the study and had usable data: HIV-/Smok- (*n* = 16), HIV-/Smok+ (*n* = 14), HIV+/Smok- (*n* = 18), and HIV+/Smok+ (*n* = 11). Following telephone screening, participants underwent an intake visit, cognitive assessment with the 5C-CPT and [^18^F]FEPPA PET/computed tomography (CT) scanning in the same session, and a structural magnetic resonance imaging (MRI) scan. The study was approved by local IRBs at UC San Diego (#170943) and the VA San Diego Healthcare System (#1198260), and informed consent was obtained from all participants.

### Intake visit—comprehensive neurobehavioral and neuromedical characterization

#### Inclusion/exclusion criteria

Participants had an intake visit to determine if they met inclusion/exclusion criteria (see Supplemental Information for details). Potential participants were included if they were adults with documented HIV status who were daily cigarette smokers or non-smokers (either never smokers or >1 year abstinent). Exclusion criteria were any major recent psychiatric diagnosis, history of conditions that could affect the CNS at scanning other than HIV, daily anti-inflammatory use, unstable medical conditions, and pregnancy. Occasional drug/alcohol use not meeting criteria for abuse/dependence was not exclusionary, but participants were instructed to abstain from use for >24 h prior to PET/CT scanning.

#### Participant assessments and interview

Rating scales were administered during the intake visit for basic background and symptom information, including the Smoker’s Profile Form [40] (demographic, race/ethnicity, educational level, medications, smoking history, and other information), Fagerström Test for Nicotine Dependence [46, 47] (FTND; severity of Nicotine Dependence), Composite International Diagnostic Interview [48] (CIDI; psychiatric characteristics), Beck Depression Inventory II (BDI) [49], and Profile of Mood States (POMS) [50]. Due to the COVID-19 pandemic, alternative arrangements were made to obtain some scales remotely during part of the study.

#### HIV disease characteristics

HIV serostatus was determined via self-report and a finger stick point-of-care test (MedMira Inc., Nova Scotia, Canada). Intake information included self-report of estimated date of HIV diagnosis, nadir CD4+ cell count (for participants where laboratory values were unavailable), list of ARTs, and AIDS status (based on conditions that determine this diagnosis). One participant’s nadir CD4+ count was unavailable by these methods. HIV RNA levels in plasma were measured via reverse transcriptase-polymerase chain reaction (Amplicor, Roche Diagnostics, Indianapolis, IN) and were considered undetectable below the lower limit of quantitation of 50 copies/ml.

#### TSPO genotyping

Prior research demonstrates that genotyping can determine an individual’s TSPO affinity subtype (high, medium, and low), and that these affinities affect binding for radioligands determining TSPO availability [51, 52]. Genotyping of rs6971 within the TSPO gene was performed by polymerase chain amplification using buccal cells (see Supplemental Information). Only participants with the high or medium affinity genotypes (>90% of North Americans [53]) were included in PET/CT data analysis to avoid a potential confound.

### 5-choice continuous performance test (5C-CPT), cognitive control assessment

**Fig. 1:**
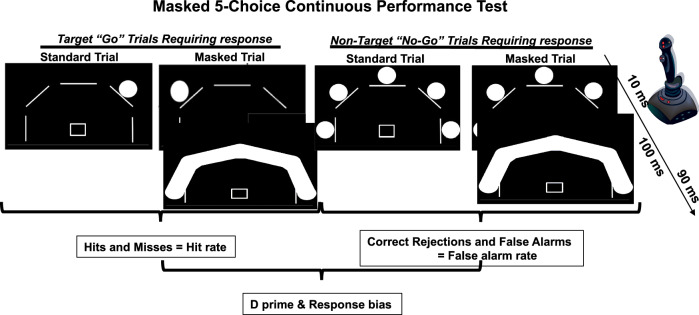
Schematic of example trials in the masked 5-choice continuous performance test. The masked 5C-CPT sequentially presents the participant with target (*n* = 180; single circle) and non-target (*n* = 36; all five circles) trials (0.5–1.5 s between trials). Participants should respond (hit) or inhibit from responding (correct rejection) to these stimuli respectively. All trials have stimuli that last 100 ms, but half are randomly presented as: (1) standard trials with stimuli appearing for 100 ms, or; (2) masked trials with stimuli presented for 10 ms prior to a mask being placed over the stimuli. The inclusion of such masks require participants to focus their attention throughout testing. Responses are made via a joystick. Data from target trials (hits or misses) are used to generate hit rates, while data from non-target trials (correct rejections and false alarms), are used to generate the false alarm rate. Signal detection theory analyses of hit and false alarm rates generate measures of d prim and responses bias.

On the PET/CT scanning day, participants arrived at the UCSD PET/CT Center at midday and the 5C-CPT [54] was administered prior to scanning. Smokers were instructed to smoke as per their typical use on the day of scanning and smoked to satiety prior to test administration. For the 5C-CPT, participants were instructed to move a joystick toward a single white circle (target trials) and inhibit from responding when all 5 circles turned white (non-target trials) (Fig. 1). Two different conditions of target and non-target stimuli were used where unmasked condition or “easy” stimuli were presented for 100 ms, and masked condition or “hard” stimuli were presented for 10 ms [55]. During the masked condition, a solid white mask was presented over the stimulus array for 90 ms after initial stimulus presentation (100 ms total, consistent with standard trials). All stimuli were presented in a pseudorandom order so that no more than 3 of the same trial types appeared consecutively, with a 1 s response window available for all trials and a variable inter-trial interval (ITI; 500, 1000 or 1500 ms). To demonstrate understanding of the procedure, all participants correctly performed a practice run prior to testing. The full task consisted of 216 trials, 108 unmasked (90 target and 18 non-target stimuli) and 108 masked (90 target and 18 non-target stimuli). Several participants did not complete this task due to fatigue.

Responses were recorded for targets (hits and misses) and non-targets (false alarms and correct rejections). Composite metrics of task performance were used in the analysis of performance, including hit rate, false alarm rate, and accuracy (hit/responses to non-lit locations), hit reaction time, all compiled by trial condition (unmasked or “easy” vs. masked or “hard”). The d prime and responsivity indices (measures of vigilance and bias, respectively) were calculated using signal detection theory, for appropriate responding and response bias, respectively.

### PET/CT session measurements—biological measures and symptom questionnaires

Following the 5C-CPT, pre-PET/CT measures were obtained (see Supplementary Information for details). Exhaled CO level was measured immediately prior to PET/CT scanning (after smoking to satiety as described in the next section) to verify smoking status. In addition, a urine cotinine screen, breathalyzer, urine toxicology screen, height, weight, and urine pregnancy test (for female participants of childbearing potential) were also obtained. Given that roughly 18% of American adults use marijuana [56] and that urine toxicology screens may remain positive for 3–7 days with a single use [57], participants were not excluded for a positive urine toxicology screen for marijuana or other recreational drugs, but were instructed to abstain for at least 24 h prior to scanning and verbally confirmed this point at the testing session. Before and after PET/CT scanning, state rating scales were also administered, namely the Minnesota Tobacco Withdrawal Scale (MTWS) [58] and Urge to Smoke (UTS) Scale [59].

### PET/CT and MRI scanning protocols

For the PET/CT scan, participants had an intravenous line placed by a nuclear medicine technician in a room adjacent to the scanner prior to scanning. In addition to the smoking schedule described above, participants who were smokers smoked to satiety in an outdoor area adjacent to the PET/CT scanner during the 10 min prior to scanning (18 of 25 smokers smoked 1 cigarette [range, 0.5–2.0 cigarettes]). Participants were then positioned on the PET/CT scanner and received a bolus injection of ~185 MBq of [^18^F]FEPPA, followed by a dynamic PET scan of the brain for 90 min. Study investigators obtained an IND approval from the FDA (#141607) for the use of [^18^F]FEPPA for this study, and [^18^F]FEPPA was synthesized using an established method [9, 60]. No significant between-group difference was present for injected radioligand dose (range of group means 188.6–192.9 MBq; *p* = 0.60). A whole brain structural MRI scan was obtained within a few weeks of PET/CT scanning to aid in localization of anatomical regions on the PET/CT scans (see Supplemental Information for scanning details).

### PET/CT and MRI scan analysis

Similar to previous research, [37, 38, 40, 41, 61–64] PET-to-MRI co-registration was performed using PMOD quantitative parametric mapping software (Bruker, U.S.), and automated volumes of interest (VOIs) were determined on MRI and transferred to co-registered PET/CT scans. The primary VOI was WB determined with FSL FAST. Since automated volumes were easily obtained and regional differences were possible, VOIs were also determined for the amygdala, caudate, globus pallidus, hippocampus, nucleus accumbens, putamen, and thalamus.

To obtain a quantitative measurement of VOI binding of TSPO in brain, SUVs were calculated using the standard definition: SUV = mean tissue activity concentration (Bq/mL)/(injected dose [Bq]/body weight [g]). Mean tissue activity concentration from 18.5 to 80 min post-injection was used, based on time-activity curves from this study demonstrating relatively stable radioligand levels during this period.

### Statistical analysis

Statistical analyses were performed on PET/CT and cognitive outcomes to test study hypotheses using SPSS version 29.0 (SPSS Inc., Chicago, IL), with WB SUVs and d prime as the primary outcome variables. A priori hypotheses were tested first, comparing WB SUV and d prime scores of HIV-/Smok- vs. HIV+/Smok- to confirm HIV-related deficits, as well as HIV+/Smok- vs. HIV+/Smok+ to determine if metrics were better for PLWH that smoke cigarettes, and finally HIV-/Smok- vs. HIV-/Smok+. Study groups were then compared across other metrics using 2-way analyses of variance (ANOVAs), with HIV and smoking statuses as between-subjects factors and genotype (for the PET/CT outcome) as a nuisance covariate. Statistical analyses with the same structures as the 2 × 2 analyses above were performed for the smaller subcortical VOIs and secondary 5C-CPT variables. While we anticipated that results for the subcortical VOIs would be similar to WB results (and to one another due to values being highly intercorrelated), we ran these analyses because there was a possibility of regional differences and the data could be useful for future research using similar methods. Chi-square or Fisher’s Exact tests (for categorical variables) and *t* tests (for continuous variables) were used to compare variables relevant to only two study groups (i.e., the two groups of smokers and the two groups of PLWH). Alpha was set to *p* < 0.05, while trend effects were considered as *p* < 0.1. Exploratory correlations were determined between both WB SUV and d prime versus smoking- and HIV-related variables (along with ratings/tests done at the PET/CT session) within the groups of smokers and PLWH, respectively. Correlations were determined controlling for study group and genotype (for the PET/CT data). Variables for the correlational analyses were cigarettes per day, FTND score, duration of smoking history (years), exhaled CO level, urinary cotinine levels, duration of HIV, nadir CD4+ count, mean MTWS score, and mean UTS score. For completeness, we also determined correlations between WB SUV and 5C-CPT outcomes in the whole study sample and HIV+ groups.

## Results

### Demographics, rating scales, and other baseline variables

Study groups were well-matched for almost all variables (Table 1), with no significant differences in age, sex, race/ethnicity, education (years), height, weight, handedness, or TSPO affinity genotype (*p’s* = 0.10–0.96). For comparisons of the two Smok+ groups (HIV- vs. HIV+), no significant differences were found in cigarettes per day, FTND scores, duration of smoking, exhaled CO levels, or urinary cotinine levels (*p’s* = 0.17–1.00). For comparisons of the HIV+ groups (Smok+ vs. Smok-), no significant differences were found for nadir CD4+ cell count, percent currently using ART, or percent with AIDS (*p’s* = 0.14–0.96); however, a significant between-group difference was found in duration of HIV infection (*p* < 0.05). Therefore, duration of HIV infection was used as a nuisance covariate in comparisons between the two HIV+ groups. All alcohol breathalyzer and urine pregnancy tests were negative. The groups did not significantly differ in percentage of participants with positive urine toxicology screens for marijuana (n = 2–3/group; *p* = 0.71) and all participants reported no drug use for >24 h prior to PET/CT scanning.

**Table 1:** Demographic and health-, smoking-, and HIV- related variables for the four study groups.

Variable	HIV-/Smok- Group (*n* = 16)	HIV-/Smok+ Group (*n* = 14)	HIV+/Smok- Group (*n* = 18)	HIV+/Smok+ Group (*n* = 11)
**Demographic**				
Age	51.4 ( ± 16.0)	53.8 ( ± 13.7)	58.8 ( ± 10.9)	55.5 ( ± 9.6)
Sex (% Female)	43.4	7.1	16.7	27.3
Race/Ethnicity (%)				
African American	18.8	35.7	22.2	27.3
Hispanic	37.5	28.6	33.3	27.3
White	43.8	35.7	44.4	45.5
Education (years)	15.0 ( ± 2.1)	14.5 ( ± 3.3)	14.8 ( ± 2.5)	14.0 ( ± 1.3)
**Health-related**				
Height (Inches)	68.0 ( ± 4.3)	69.6 ( ± 3.2)	68.6 ( ± 3.2)	67.8 ( ± 4.8)
Weight (Kilograms)	79.4 ( ± 15.1)	89.5 ( ± 17.5)	91.7 ( ± 20.6)	80.2 ( ± 15.3)
Handedness (% Left)	6.3	7.1	5.6	9.1
TSPO (% High Affinity)	68.8	64.2	61.1	63.6
**Smoking-related**				
Cigarettes per day	n/a	11.7 ( ± 5.4)	n/a	18.9 ( ± 18.3)
Fagerström Test for Nicotine Dependence	n/a	4.2 ( ± 2.0)	n/a	3.4 ( ± 2.2)
Years Smoking	n/a	33.0 ( ± 15.9)	n/a	33.0 ( ± 16.9)
Exhaled Carbon Monoxide (ppm)	n/a	14.4 ( ± 7.7)	n/a	16.5 ( ± 10.0)
Urinary Cotinine Test	n/a	3.9 ( ± 1.8)	n/a	4.2 ( ± 2.0)
**HIV-related**				
Est. Duration of HIV (yrs)	n/a	n/a	22.1 ( ± 10.3)*	13.3 ( ± 7.6)
Nadir CD4+ Cell Count (cells/µL)	n/a	n/a	288 ( ± 331)	312 ( ± 311)
Current ART Use (%)	n/a	n/a	100	82
AIDS (%)	n/a	n/a	55.6	54.5

*TSPO* translocator protein, *ppm* parts per million, *ART* antiretroviral therapy.

All values are presented as means (± standard deviation) or percentages. Using analyses of variance and Student *t* tests for continuous variables and χ^2^ tests for categorical variables, no between-group tests were significant, other than the difference in duration of HIV between the two HIV+ groups (*p < 0.05).

### Effect of HIV and Smoking Statuses on PET/CT Findings

**Fig. 2:**
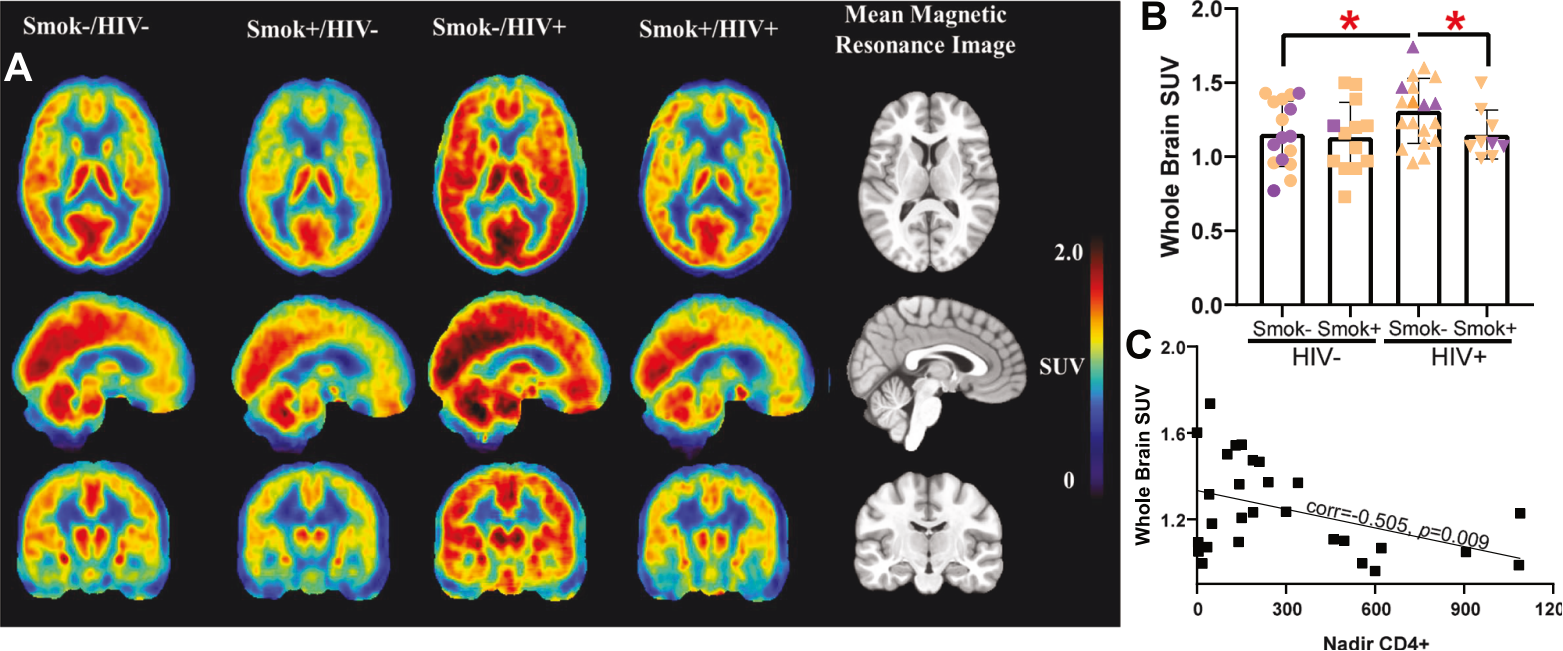
PLWH that smoke cigarettes have reduced levels of the marker for neuroinflammation relative to their non-smoking counterparts. Mean PET/CT images from participants who do not have HIV (HIV-) vs. those that do (HIV+), and those that smoke cigarettes (Smok+) vs. those that do not (Smok-). As can be seen, the highest (brightest red) levels of the marker for neuroinflammation were seen in HIV+Smok- participants, while PLWH who smoke had reduced levels (HIV+Smok+; **A**). These levels were quantified and found to be significant (**B**). Levels of neuroinflammation negatively correlated with nadir CD4+ levels (**C**). *=*p* < 0.05 as indicated.

The overall analysis of PET/CT data revealed a significant effect of smoking status (F[1, 54] = 7.7, *p* = 0.008) and a trend-level effect of HIV status (F[1, 54] = 2.8, *p* = 0.098), with no significant interaction between smoking and HIV statuses (F[1, 54] = 1.5, *p* = 0.22). The ranking of group mean WB SUVs was consistent with the hypothesized effects of HIV increasing and smoking decreasing radioligand binding (Table 2; Fig. 2). In examining the specific contrast for the primary a priori hypothesis, a significant effect of smoking status on WB SUV was found in PLWH (F[1, 25] = 4.9, *p* < 0.05) due to HIV+/Smok+ having a mean 18.8% lower WB SUV than HIV+/Smok-. Controlling for age in the model did not significantly change the observed difference between the HIV+/Smok- and HIV+/Smok+ groups (F[1, 24] = 4.9, *p* < 0.05), indicating that age was not a confounding factor for the effect of smoking status on WB SUV in PLWH. No between-group differences were found between HIV- smokers and non-smokers (F[1, 28] = 1.0, ns), though smokers had a mean 8.0% lower WB SUV than non-smokers. HIV+/Smok- exhibited higher WB SUV than HIV-/Smok- individuals (F[1, 31] = 4.7, *p* < 0.05), with a mean 13.5% higher WB SUV in non-smoking PLWH.

**Table 2:** Standardized uptake values (SUVs) for the whole brain and smaller regions of interest for the four study groups.

Brain Region	HIV-/Smok- Group (*n* = 16)	HIV-/Smok+ Group (*n* = 14)	HIV+/Smok- Group (*n* = 18)	HIV+/Smok+ Group (*n* = 11)
Whole Brain	1.17 (±0.22)	1.08 (±0.25)	1.34 (±0.22)^a^	1.11 (±0.11)
Accumbens R L	1.31 (±0.27) 1.32 (±0.25)	1.25 (±0.36) 1.22 (±0.34)	1.52 (±0.38)^b^ 1.50 (±0.39)^b^	1.24 (±0.15) 1.22 (±0.11)
Amygdala R L	1.18 (±0.23) 1.17 (±0.23)	1.07 (±0.26) 1.05 (±0.25)	1.32 (±0.30)^a^ 1.30 (±0.27)^a^	1.09 (±0.11) 1.09 (±0.11)
Caudate R L	1.20 (±0.27) 1.20 (±0.24)	1.08 (±0.32) 1.09 (±0.29)	1.36 (±0.35)^b^ 1.32 (±0.42)	1.04 (±0.31) 1.15 (±0.15)
Globus Pallidus R L	1.08 (±0.24) 1.08 (±0.23)	0.98 (±0.27) 1.02 (±0.27)	1.27 (±0.36)^b^ 1.28 (±0.31)^b^	1.05 (±0.14) 1.02 (±0.12)
Hippocampus R L	1.29 (±0.25) 1.27 (±0.18)	1.16 (±0.30) 1.18 (±0.29)	1.44 (±0.27)^a^ 1.46 (±0.26)^b^	1.20 (±0.16) 1.20 (±0.15)
Putamen R L	1.46 (±0.31) 1.46 (±0.30)	1.36 (±0.36) 1.35 (±0.35)	1.62 (±0.43) 1.65 (±0.41)	1.39 (±0.15) 1.41 (±0.15)
Thalamus R L	1.48 (±0.29) 1.45 (±0.29)	1.38 (±0.41) 1.34 (±0.39)	1.73 (±0.37)^b^ 1.70 (±0.37)^b^	1.42 (±0.16) 1.42 (±0.16)

All values are mean ± standard deviation. R = right, L = left. All regions were analyzed using an overall analysis of variance, with HIV and smoking statuses as between-subject factors and genotype as a nuisance covariate. Automated regions listed here were generated using the FSL toolkit.

^a^*p*  ≤ 0.01, for overall effect of smoking status.

^b^*p*  < 0.05, for overall effect of smoking status.

Consistent with the WB findings, SUVs for eleven of the fourteen smaller VOIs had a significant effect of smoking status (F’s[1, 54] = 4.2–7.6, *p’s* = 0.008–0.04), while the remaining VOIs approached significance (F’s[1, 54] = 2.8–3.8, *p’s* = 0.06–0.10). Also similar to the WB findings, three of the fourteen smaller VOIs had effects of HIV status that approached significance (F’s[1, 54] = 2.8–3.7, *p’s* = 0.06–0.10), while the remaining VOIs did not reach significance (F’s[1, 54] = 0.5–2.5, *p’s* = 0.12–0.48). These smaller VOIs had a similar order of SUVs as for WB SUVs listed above (Table 2). Subcortical VOI SUVs were highly intercorrelated (all *p*’s < 0.001) and the findings above indicate no significant regional differences.

In the exploratory analyses of smoking- and HIV- related variables, a significant inverse relationship was found between WB SUV and nadir CD4+ levels (partial correlation coefficient = –0.51, *p* = 0.009). Two smoking-related variables (urinary cotinine and mean UTS scores) had inverse associations with WB SUV that approached significance (partial correlation coefficients = –0.41, *p* = 0.06 and –0.38, *p* = 0.08, respectively). The remaining variables did not significantly correlate with WB SUV (*p’s* = 0.11–0.56).

### Smoking status was associated with better 5C-CPT performance

**Fig. 3:**
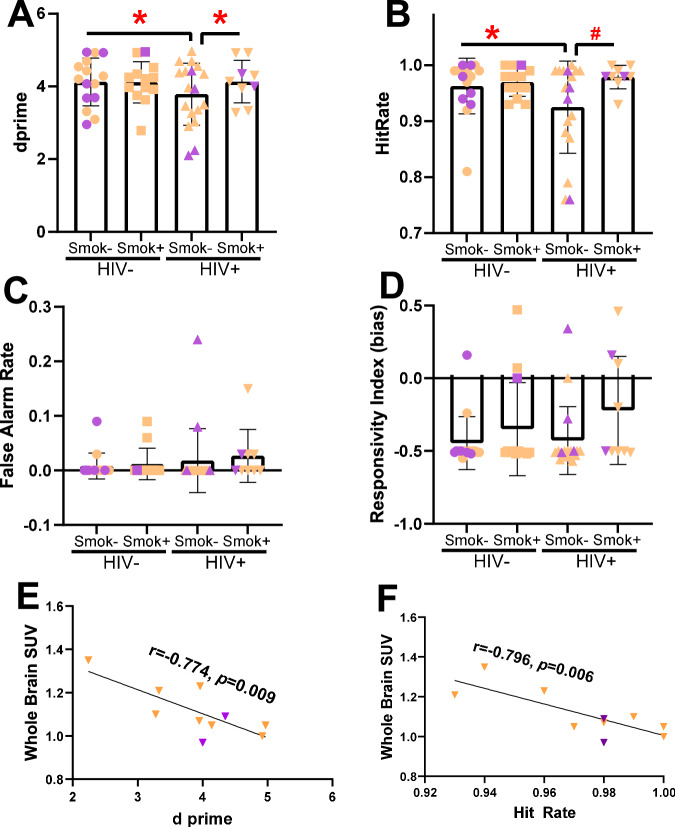
PLWH that smoke (Smok+) cigarettes performed the masked 5C-CPT better than PLWH who do not smoke (Smok-). Overall performance was measured using d prime, which revealed that seronegative individuals (HIV-) performed the masked 5C-CPT better than PLWH (HIV+; **A**). Importantly however, PLWH that smoke exhibited better performance than PLWH non-smokers (**A**). This effect was driven by improved target detection in Smok+ HIV+ individuals as measured by Hit Rate (**B**). No effect of HIV or smoking status was seen in response disinhibition as measured by false alarms (**C**), or response bias (**D**). The WB SUV measurement of neuroinflammation correlated negatively with d prime (**E**) and hit rate (**F**) in HIV+/Smok+ participants. Data presented as individual data points, means ± .S.E.Ms. Purple = female, orange = male. *denotes *p* < 0.05 as indicated, *#*denotes *p* < 0.1 as indicated.

Consistent with our primary a priori hypotheses, HIV seropositivity had a detrimental effect on overall performance (d prime) in non-smokers. HIV+/Smok- participants tended to perform worse on d prime than HIV-/Smok- (F[1, 27] = 3.5, *p* = 0.07; Fig. 3). Importantly, within PLWH, smoking status was associated with a trend toward better d prime; HIV+/Smok+ exhibited higher scores than HIV+/Smok- (F[1, 27] = 3.1, *p* = 0.09; Fig. 3). Adding age to this model as a covariate did not alter results. Thus, smoking in PLWH was associated with 5C-CPT performance comparable to HIV-/Smok- participants (*p* > 0.1).

Secondary analyses revealed the potential mechanism underlying these smoking-associated improvements. Smokers tended to have increased target detection (hit rate) (F[1, 50] = 3.5, *p* = 0.07), with no effect on response inhibition (false alarm rate) (F[1, 50] = 0.4, *p* = 0.51). In contrast, the overall poorer performance of PLWH as measured by d prime was not specifically driven by poorer target detection (F[1, 50] = 1.1, *p* = 0.31) or non-target responding (F[1, 50] = 2.4, *p* = 0.13). Consistently, no smoking/HIV status interaction was observed for hit rate (F[1, 50] = 0.9, *p* = 0.35) or false alarm rate (F[1, 50] = 0.9, *p* = 0.35). As with d prime, the effects of smoking arose because smoking status impacted performance in PLWH whereby HIV+/Smok+ participants had a higher hit rate than HIV+/Smok- participants (*p* < 0.05). No effect of smoking status was seen among HIV- participants. This former observation was likely due to the trend poorer hit rate in HIV+/Smok- relative to HIV-/Smok- participants (*p* = 0.06), indicating the impact of HIV on target detections amongst non-smokers.

While the impact of masking trials in the 5C-CPT negatively affected every measure, including d prime (F[1, 50] = 62.8, *p* < 0.001), hit rate (F[1, 50] = 22.7, *p* < 0.0001), and false alarm rate (F[1, 50] = 5.6, *p* < 0.05), no interaction between mask condition, smoking, or HIV status was observed (Fs < 1.8), except a smoking*mask interaction on hit rate (F[1, 50] = 4.3, *p* < 0.05). Post hoc analyses revealed that Smok+ participants exhibited better target detection relative to Smok- participants during mask trials (*p* < 0.05). Other neuropsychological test findings support modest positive effects of cigarette smoking on some test performance (see Supplemental Information).

### Correlations between WB SUV and 5C-CPT outcomes

No significant correlations were observed in the whole group between WB SUV and the 5C-CPT main outcomes (d prime, hit rate, and false alarm rate; *p’*s = 0.36–0.97). However, within the HIV+/Smok+ group, significant inverse correlations were found between WB SUV and both d prime (r = –0.77, *p* = 0.009) and hit rate (r = –0.80, *p* = 0.006), indicating that less neuroinflammation in HIV+ smokers is associated with better cognitive control in this group (Fig. 3).

## Discussion

Here, we present evidence that cigarette smoking is associated with reduced neuroinflammation and better cognitive control in PLWH. Specifically, PLWH who smoke cigarettes had lower levels of the PET/CT neuroinflammation marker and better cognitive control on neuropsychological testing than PLWH non-smokers. These data are in agreement with prior findings that, compared to non-smoking healthy participants, non-smoking PLWH have higher levels of TSPO throughout the brain and poorer cognitive control. The cognitive control enhancing effects of smoking appear driven by increasing target detection, particularly in more difficult masked trials. In addition, an exploratory analysis indicated that PLWH with lower nadir CD4+ levels had higher levels of the neuroinflammation marker. Furthermore, while a direct correlation between WB SUV and d prime in the 5C-CPT was not found for the whole study group, significant correlations were seen specifically in PLWH who smoked (as well as in hit rate); therefore, it is reasonable to hypothesize that nicotine provides benefit for the negative effects of chronic HIV infection on cognitive control, possibly through effects on neuroinflammation. Taken together, these data indicate potential positive effects of cigarette smoking on neuroinflammatory and cognitive processes in PLWH.

As for effects of smoking on HIV-associated neuroinflammation, HIV enters the brain early after infecting humans and may remain in the CNS despite successful ART [65]. As noted above, PET studies similar to this one demonstrated that PLWH have elevated levels of the PET marker for neuroinflammation and higher levels of the marker are associated with worse cognitive impairments [19, 21]. Based on this (and related) research, it has been hypothesized that ongoing chronic neuroinflammation contributes to cognitive impairment in otherwise effectively-treated PLWH [66]. Recent research demonstrates that nicotine exerts much more anti- than pro-inflammatory effects [6] and a recent review of animal literature in HIV and smoking concluded that preclinical studies using nicotine show mild cognitive enhancement, neuroprotective, and possibly anti-inflammatory effects [26]. This premise is particularly supported in the present study by the negative correlations between the marker for neuroinflammation and both d prime and hit rate measurements from the 5C-CPT in PLWH who smoke. Of course, the known harmful effects of chronic smoking [67] far outweigh any potential benefits found here, such that this study should not be interpreted as a reason for continued smoking in PLWH.

Study results provide additional evidence that PLWH have chronic neuroinflammation and that cigarette smoking results in lower levels of the marker for neuroinflammation. These results are in agreement with prior research in PLWH [19, 21], even though a different PET outcome measure and radioligand were used. Similarly, study results were generally consistent with prior PET studies of cigarette smoking and TSPO availability in people without HIV [37, 38], supporting the evidence that smoking leads to lower levels of neuroinflammation.

HIV has long been associated with cognitive deficits, particularly in cognitive control and attention [68]. While some computerized tests of attention have not revealed deficits [69, 70], the current methods utilized backward masks to increase difficulty, revealing deficits in PLWH. Importantly, smoking was associated with improved overall cognitive control/attentional performance increasing target detection (hit rate), with no effect on non-target responding (false alarm rate), consistent with chronic nicotine administration in rodents [27]. Relatedly, chronic nicotine patch improved attention in healthy participants [28] and people with Attention Deficit Hyperactivity Disorder [71], supporting the broad attention-enhancing effects of nicotine treatment which may be associated with improved cognitive control in PLWH. Furthermore, the masking condition in this 5C-CPT may have made performance sufficiently difficult to enable observation of both deficits and improvements. These data contrast with prior studies observing poorer cognitive performance in PLWH that smoke relative to their non-smoking counterparts [30–32]; however, in the current study, we accounted for the potential confound of individuals undergoing withdrawal by testing soon after smoking to satiety [33]. A trend interaction was observed between PLWH and smoking on verbal fluency, with no effects on other domains (see Supplemental Fig. 1). With better 5C-CPT performance in smokers, one may have anticipated improved executive functioning, but it could be that the computerized testing was more demanding than such paper and pen tests, hence smoking effects in the former and not the latter. This work may confirm why some studies of chronic nicotine/tobacco use show beneficial effects in animals and humans (computerized testing plus smoking satiety), while others do not (use of paper and pen tests and testing during withdrawal).

As for exploratory correlations, the inverse association between nadir CD4+ cell count and WB SUV indicates that a history of severe HIV infection results in greater neuroinflammatory processes (as previously proposed [72]). Consistent with this idea, nadir CD4+ count is a factor that predicts cognitive impairment in PLWH [73] and modifies ART magnitude effects on cognitive outcomes [74]. As for the smoking-related variables, the negative associations (approaching significance) between urinary cotinine (and craving) scores and WB SUV are consistent with prior research [37, 38] whereby variables indicating greater cigarette smoke exposure were associated with lower levels of the neuroinflammation marker. The trend-level negative association between urinary cotinine and WB SUV is consistent with much prior research demonstrating that cotinine reduces neuroinflammation [75, 76].

This study should be interpreted in the context of several limitations. Because cigarette smoke contains thousands of constituents [77, 78], it is not possible to know which resulted in the lower radioligand binding levels and better cognitive control found here, although given similar effects of nicotine described above, it is likely a factor. Also, while radioligand binding to TSPO is substantially related to its expression during microglial activation, TSPO has other locations which could affect the interpretation of study results [15, 79, 80]. Additionally, the absence of arterial blood sampling precluded determination of total distribution volume (Vt), a gold standard outcome measure for this type of research. Vt may control for potential confounds of between-subject differences in radioligand metabolism and binding to vascular endothelium and plasma protein [81, 82]. While Vt is a common PET outcome measure, SUV was used here because it avoids invasive arterial blood sampling and arterial sampling was not feasible at our site. In addition, SUV has been shown in rodents to strongly correlate with Vt [83, 84], has good test-retest reproducibility [84], and has less intersubject variability than that for Vt [83] with a similar radioligand. Other similar studies have used pseudo-reference regions for PET analysis, [14, 20, 85–88] but this method would not have been appropriate due to the hypothesized and confirmed effect of HIV and smoking throughout the brain regions tested. In addition, the modest sample size may have been responsible for the whole group interaction between the PET/CT and 5C-CPT findings not being significant and the WB SUV findings not being significantly different between HIV- smokers and non-smokers as was found in prior research [37, 38], though it should be noted that a mean group difference (8.0%) between the HIV- smoker and non-smoker groups was found in the expected direction. Finally, the cross-sectional nature of the study did not allow for disentangling the effects of smoking from other factors (e.g., personality traits) that would lead someone to become dependent on tobacco.

Despite the above limitations, the study has several characteristics that indicate that findings are generalizable to PLWH who are non-smokers and smokers. First, results were consistent with prior brain imaging studies that independently examined effects of HIV [19–21] and cigarette smoking [37, 38] statuses on PET markers for neuroinflammation, along with studies of the effects of nicotine on cognitive control [27, 39]. Second, the study population was racially diverse (Table 2) consistent with the diverse population of San Diego County. And third, cigarette smoking prior to PET scanning and cognitive testing simulated real-world smoking in that participants smoked to satiety in an outdoor area, which was intended to enhance generalizability.

In summary, while PLWH exhibited elevated neuroinflammation relative to people without HIV, PLWH who smoke had diminished neuroinflammation and improved cognitive control compared to PLWH non-smokers. This study indicates that nicotine could be beneficial for conditions mediated by chronic neuroinflammation such that delineating the neural mechanism mediating these effects could lead to targeted therapeutics for both neuroinflammation and cognitive symptoms in PLWH.

## Supplementary information

Supplemental Material

## Data Availability

De-identified participant data are available upon request.
