# Supplementary material for: Cigarette smoking is associated with reduced neuroinflammation and better cognitive control in people living with HIV
Source: Neuropsychopharmacology. 2025 Jan 1;50(4):695–704. doi: 10.1038/s41386-024-02035-6 (PMC11845771; doi:10.1038/s41386-024-02035-6)
Supplement: Supplementary file 1 — Supplemental Material [file 41386_2024_2035_MOESM1_ESM.pdf]

Supplemental Information

Inclusion/Exclusion Criteria: Participants had an intake visit to determine if they met inclusion/exclusion criteria. Inclusion Criteria were: (1) adults (18-75 years old) with documented HIV status who were daily cigarette smokers meeting criteria for Tobacco Use Disorder(1) or non-smokers (never users or >1 year tobacco free), (2) ability to read, write, and provide informed consent, and (3) confirmation of smoking status through exhaled CO levels (≥8 ppm for smokers, <8 ppm for non-smokers). Exclusion criteria were: (1) any major psychiatric diagnosis (including mood, anxiety, psychotic, and substance use disorders) within the past year. Occasional drug/alcohol use not meeting criteria for abuse/dependence was not exclusionary, but participants were instructed to abstain from use for >24 h prior to PET/CT scanning, (2) history of conditions that could affect the CNS at scanning (e.g., severe head trauma, epilepsy, or other neurological diseases) other than HIV, (3) daily anti-inflammatory use, (4) unstable medical disease which might make tolerating procedures difficult, and (5) pregnancy.

TSPO Genotyping: DNA was extracted from participants' buccal cells collected at intake using the Oragene OG-500 kit (DNA Genotek; Ottawa, Ontario, Canada) according to manufacturer protocol. Genotyping of rs6971 within the TSPO gene was performed by polymerase chain amplification (Forward Primer: 5'- AAGCGTGACGGCCACCACATCA – 3'; Reverse Primer: 5'-CCTGACTCCCAAATCCAGTG-3') of a 362 base pair fragment of the *TSPO* gene containing the rs6917 followed by restriction enzyme digestion with *NruI* (New England Biolabs; Ipswich, MA) in the laboratory of a study co-investigator (B.R.).

Testing at the PET/CT Session: Prior to scanning, exhaled CO level was measured with the Micro+ Smokerlyzer Breath CO Monitor (Bedfont Scientific, Ltd, UK) immediately prior to PET/CT scanning, with CO levels of $\geq$8 ppm consistent with recent smoking, <8 ppm consistent with non-smoking status. The Accutest® NicAlert™ (Jant Pharmacal Corp., Encino, CA) immunoassay urine cotinine screen assessed recent nicotine exposure on the PET/CT scanning day, with levels $\geq$3 (100-200 ng/mL) indicating recent use of tobacco products. Both a breathalyzer (AlcoMatePro) and urine toxicology screen (Test Country I-Cup Urine Toxicology Kit) were obtained at the baseline visit and at the PET/CT scanning session. Given that roughly 18% of American adults use marijuana(2) and that urine toxicology screens may remain positive for 3-7 days with a single use(3), participants were not excluded for a positive urine toxicology screen for marijuana or other recreational drugs, but were instructed to abstain for at least 24 h prior to PET/CT scanning and verbally confirmed this point at the testing session. Height and weight were also obtained, and a urine pregnancy test (β-HCG) (Test Country Cassette Urine Pregnancy Test) was obtained for all female participants of childbearing potential.

State Rating Scales obtained at the PET/CT Session: Before and after PET/CT scanning, state rating scales were administered, namely the Minnesota Tobacco Withdrawal Scale (MTWS)(4) and Urge to Smoke (UTS) Scale(5). The MTWS-revised comprehensively assessed cigarette withdrawal symptoms (including irritability, anxiety, depression, and craving). The UTS obtained ratings from 1 to 7 (definitely not to definitely) on craving-related items, and analog ratings of mood and anxiety were single items ranked from 1 to 7 (very bad to very good and none to extremely strong, respectively).

PET/CT and MRI Scanning: PET/CT scans were obtained using a G.E. Healthcare Discovery MI DR PET/CT scanner (G.E. Healthcare Co.) in three-dimensional (3D) mode, with CT

scanning for attenuation correction. Iterative image reconstruction was performed, resulting in scans with 47 transaxial slices, reconstruction diameter 30 cm, and a pixel size of approximately 1.17 mm x 1.17 mm. A structural whole brain MRI scan was obtained using established protocols and co-registered with the PET/CT scans to aid in localization of anatomical regions on the PET/CT scans. Whole brain T1- and T2-weighted images were obtained on a 3.0T GE MR750 scanner (GE Medical Systems, Milwaukee, WI) with 0.8mm slice thickness with a FOV=256x240 mm: T1 MPRAGE-like SPGR sequence, TE=2.3ms, flip angle=8°, matrix=320, scan time=7:50; and T2 3D CUBE sequence with TE=60ms, TR=3200ms, ETL=200, matrix=320, scan time=6:51. The MRI procedure lasted <30 min.

Neuropsychological Assessment: Participants completed a standardized battery of 15 neuropsychological tests designed to provide a comprehensive assessment of seven cognitive domains most affected by HIV (Supplemental Table 1): verbal fluency, executive functioning, processing speed, learning, memory, attention/working memory, and fine motor skills. Individual raw scores were converted into demographically adjusted (age, sex, education, race/ethnicity) T-scores, which were averaged within each domain across the entire battery to derive mean global and domain-specific T-scores.

Supplemental Table 1: Neuropsychological battery

**Neurocognitive Domain**	**Test**
**Verbal fluency**	COWAT Category Fluency (Animals & Actions); Letter Fluency (FAS) [6,7]
**Executive functioning**	Wisconsin Card Sorting Test-64 (Perseverative responses) [8,9]<br>Trail Making Test, Part B [6], Stroop Color-Word Trial [10,11]
**Processing speed**	WAIS-III Digit Symbol Coding [6,7]; WAIS-III Symbol Search [6,7]<br>Trail Making Test, Part A [6]; Stroop Color Trial [10,11]
**Learning**	Hopkins Verbal Learning Test-Revised (Trials 1-3) [10,13]<br>Brief Visuospatial Memory Test-Revised (Trials 1-3) [10,14]
**Memory**	Hopkins Verbal Learning Test-Revised (Delayed recall) [10,13]<br>Brief Visuospatial Memory Test-Revised (Delayed recall) [10,14]
**Attention/working memory**	WAIS-III Letter-Number Sequencing [11,12]<br>PASAT (first channel only) [15]
**Motor skills**	Grooved Pegboard Test (Dominant hand, non-dominant hand) [6,16]

Note., COWA = Controlled Oral Word Association Test, WASI-III = Wechsler Adult Intelligence Scale-3rd edition, PASAT = Paced Auditory Serial Addition Task.

To determine how NCI relates to functional decline, the global deficit score (GDS) approach was utilized [17]. For greater specificity and focus on impairment, domain deficit scores (DDS) were considered as neurocognitive predictors in regression models. The GDS method assigns a deficit score to each T-score (M = 50, SD = 10), which increases with greater impairment (i.e., 0 = no cognitive impairment [T-scores ≥ 40]; 5 = severe cognitive impairment [T-scores < 20]). Deficit scores across all tests were averaged with a cut-off of GDS ≥ 0.5 indicating NCI. Deficit scores were averaged within cognitive domains to create DDS with a cut-off of DDS > 0.5 indicating NCI.

Supplemental Table 2: Impact of cigarette smoking and HIV status on neuropsychological test battery

Domain	HIV	Smoking	HIV*Smoking	Post Hoc
Global	$F_{(1,50)}=0.6$, $p=0.449$	$F_{(1,50)}=0.3$, $p=0.591$	$F_{(1,50)}=0.7$, $p=0.403$	N/A
Verbal	$F_{(1,50)}=0.4$, $p=0.506$	$F_{(1,50)}=0.9$, $p=0.350$	**$F_{(1,50)}=3.8$, $p=0.057$**	*HIV+Tob+>HIV+Tob-*
Executive	$F_{(1,50)}=0.0$, $p=0.911$	$F_{(1,50)}=0.3$, $p=0.595$	$F_{(1,50)}=0.0$, $p=0.888$	N/A
SpeedProc	$F_{(1,50)}=1.0$, $p=0.320$	$F_{(1,50)}=0.6$, $p=0.461$	$F_{(1,50)}=0.0$, $p=0.894$	N/A
Learn/Mem	**$F_{(1,50)}=3.2$, $p=0.081$**	$F_{(1,50)}=0.0$, $p=0.969$	$F_{(1,50)}=2.2$, $p=0.149$	*HIV+>HIV-*
Recall	$F_{(1,50)}=0.6$, $p=0.460$	$F_{(1,50)}=0.4$, $p=0.528$	$F_{(1,50)}=2.3$, $p=0.140$	N/A
Work Mem	$F_{(1,50)}=1.0$, $p=0.328$	**$F_{(1,50)}=6.7$, $p=0.013$**	$F_{(1,50)}=0.0$, $p=0.924$	*Tob+>Tob-*
Motor	$F_{(1,50)}=0.9$, $p=0.342$	$F_{(1,50)}=0.2$, $p=0.677$	$F_{(1,50)}=0.1$, $p=0.727$	N/A

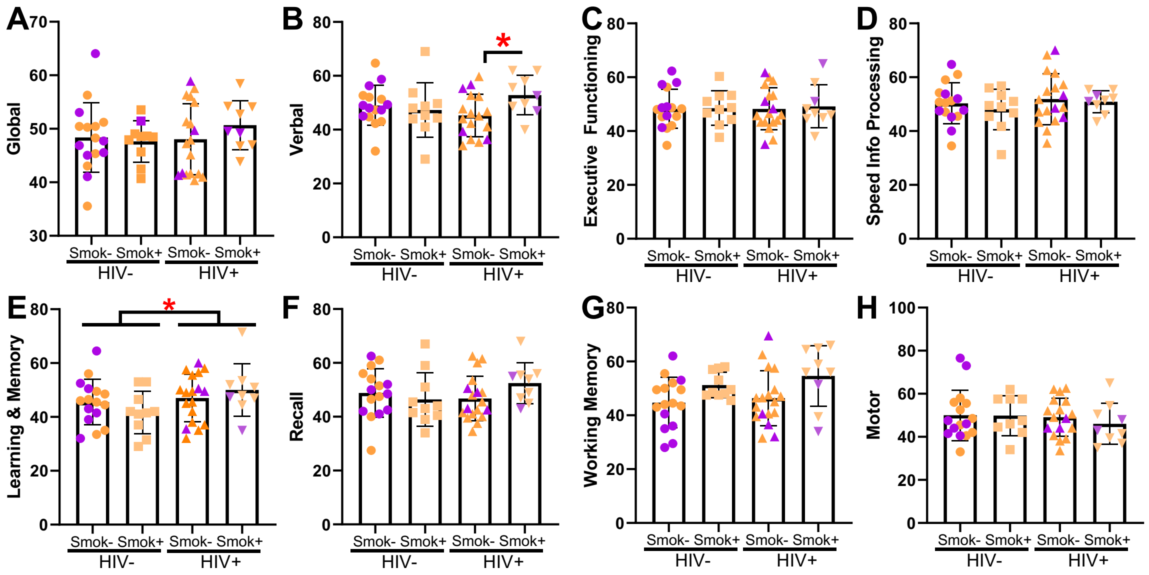

Supplemental Figure 1: PLWH that smoke (Smok+) cigarettes perform some neuropsychological tests better than non-smokers (Smok-). Global scores were unaffected by smoking or HIV status (**A**). Cigarette smoking PLWH (HIV+) exhibited higher verbal scores than non-smoking PLWH (**B**). Neither tobacco use nor HIV status affected scores of executive functioning (**C**) or speed of information processing (**D**). PLWH exhibited better learning and memory scores than seronegative (HIV-) participants (**E**), while no effect of either status affected recall scores (**F**). Cigarette smokers exhibited better working memory scores than non-smokers (**G**). Neither cigarette use nor HIV status affected motor scores (**H**). Data presented as individual data points, means, ±.S.E.Ms. Purple = female, orange = male. *denotes $p<0.05$ as indicated, # denotes $p<0.1$ as indicated.

1. AmericanPsychiatricAssociation. Diagnostic and statistical manual of mental disorders: DSM-5. Washington, D.C.: American Psychiatric Association; 2013.
2. SAMHSA. Key substance use and mental health indicators in the United States: Results from the 2019 National Survey on Drug Use and Health. In: Quality CfBHSa, editor. Rockville, MD2020.
3. Moeller KE, Kissack JC, Atayee RS, Lee KC. Clinical Interpretation of Urine Drug Tests: What Clinicians Need to Know About Urine Drug Screens. Mayo Clin Proc. 2017;92(5):774-96.

4. Hughes JR. Measurement of the effects of abstinence from tobacco: a qualitative review. Psychol Addict Behav. 2007;21(2):127-37.
5. Jarvik ME, Madsen DC, Olmstead RE, Iwamoto-Schaap PN, Elins JL, Benowitz NL. Nicotine blood levels and subjective craving for cigarettes. Pharmacology Biochemistry and Behavior. 2000;66:553-8.
6. Heaton RK, Miller W, Taylor MJ, I. G. Revised comprehensive norms for an expanded Halstead-Reitan battery: Demographically adjusted neuropsychological norms for African American and Caucasian adults, professional manual. Lutz, FL: Psychological Assessment Resources, Inc.; 2004.
7. Woods SP, Scott JC, Sires DA, Grant I, Heaton RK, Troster AI, and the HNRC Group. (2005). Action (verb) fluency: test-retest reliability, normative standards, and construct validity. Journal of the International Neuropsychological Society, 11, 408-415
8. Kongs, S. K., Thompson, L. L., Iverson, G. L., & Heaton, R. K. (1993). Wisconsin Card Sorting Test–64 card version professional manual. Odessa, FL: Psychological Assessment Resources.
9. Golden, C. J., & Freshwater, S. M. (2002). Stroop Color and Word Test: A manual for clinical and experimental uses. Lutz, FL: Psychological Assessment Resources
10. Norman MA, Moore DJ, Taylor M, Franklin D, Jr., Cysique L, Ake C, et al. Demographically corrected norms for African Americans and Caucasians on the Hopkins Verbal Learning Test-Revised, Brief Visuospatial Memory Test-Revised, Stroop Color and Word Test, and Wisconsin Card Sorting Test 64-Card Version. J Clin Exp Neuropsychol. 2011;33(7):793-804.
11. Wechsler D. Wechsler Adult Intelligence Scale – Third Edition. San Antonio, TX: Psychological Corporation; 1997.
12. Heaton, R. K., Taylor, M. J., & Manly, J. J. (2003). Demographic effects and use of demographically corrected norms for the WAIS–III and WMS–III. In D. Tulsky, D. Saklofske, G. Chlune, R. Heaton, R. Ivnik, R. Bornstein, A. Prifitera, & M. Ledbetter (Eds.), Clinical interpretation of the WAIS–III and WMS–III (practical resources for the mental health professional) (pp. 183–210). San Diego, CA: Academic Press.
13. Benedict R, Schretlen D, Groninger L, Brandt J. Hopkins Verbal Learning Test-Revised: Normative data and analysis of inter-form and test-retest reliability. The Clinical Neuropsychologist. 1998;12:43-55.
14. Benedict R. Brief Visuospatial Memory Test - Revised. Odessa, Florida: Psychological Assessment Resources, Inc.; 1997.
15. Diehr MC, Cherner M, Wolfson TJ, Miller SW, Grant I, Heaton RK, Group HNRC. The 50 and 100-item short forms of the Paced Auditory Serial Addition Task (PASAT): demographically corrected norms and comparisons with the full PASAT in normal and clinical samples. J Clin Exp Neuropsychol. 2003;25(4):571-85.
16. Kløve H. Grooved pegboard. Lafayette, IN: Lafayette Instruments; 1963.
17. Carey CL, Woods SP, Gonzalez R, Conover E, Marcotte TD, Grant I, et al. Predictive validity of global deficit scores in detecting neuropsychological impairment in HIV infection. J Clin Exp Neuropsychol. 2004;26(3):307-19.
